# Codon usage in vertebrates is associated with a low risk of acquiring nonsense mutations

**DOI:** 10.1186/1479-5876-9-87

**Published:** 2011-06-08

**Authors:** Pirmin Schmid, Willy A Flegel

**Affiliations:** 1 National Institutes of Health, Clinical Center, Bethesda, MD, USA

## Abstract

**Background:**

Codon usage in genomes is biased towards specific subsets of codons. Codon usage bias affects translational speed and accuracy, and it is associated with the tRNA levels and the GC content of the genome. Spontaneous mutations drive genomes to a low GC content. Active cellular processes are needed to maintain a high GC content, which influences the codon usage of a species. Loss-of-function mutations, such as nonsense mutations, are the molecular basis of many recessive alleles, which can greatly affect the genome of an organism and are the cause of many genetic diseases in humans.

**Methods:**

We developed an event based model to calculate the risk of acquiring nonsense mutations in coding sequences. Complete coding sequences and genomes of 40 eukaryotes were analyzed for GC and CpG content, codon usage, and the associated risk of acquiring nonsense mutations. We included one species per genus for all eukaryotes with available reference sequence.

**Results:**

We discovered that the codon usage bias detected in genomes of high GC content decreases the risk of acquiring nonsense mutations (Pearson's *r *= -0.95; *P *< 0.0001). In the genomes of all examined vertebrates, including humans, this risk was lower than expected (0.93 ± 0.02; mean ± SD) and lower than the risk in genomes of non-vertebrates (1.02 ± 0.13; *P *= 0.019).

**Conclusions:**

While the maintenance of a high GC content is energetically costly, it is associated with a codon usage bias harboring a low risk of acquiring nonsense mutations. The reduced exposure to this risk may contribute to the fitness of vertebrates.

## Background

Codon usage bias in genomes is relevant for organisms. It influences the translation speed and thus gene expression [1]. Artificially deoptimized codon usage can decrease gene expression and create an attenuated viral virulence that may be used for vaccine production [2]. HIV-1 modifies the tRNA pool of the infected cells to increase translation efficiency of its own genes [3]. Initial studies on codon usage bias were based on few genes in single species: lists of the codon usage [4], determination of the number of codons used in genes [5], and models, such as the codon adaptation index (CAI). The CAI compared the codon usage of each gene with an "optimal" codon usage, which is inferred from high-expression gene sets [6]. Whole genome sequencing data and newer algorithms have allowed researchers to overcome previous limitations, study more genes, and classify genes in more detailed categories [7]. Codon usage bias is associated with tRNA concentration [8] and also the GC content of genomes [9-12].

Loss-of-function mutations, such as nonsense mutations, are the molecular basis of many recessive disorders, conditions that stem from non-functional gene products or, in case of null alleles, a lack of gene products. Nonsense mutations cause the premature stop of translation with shortened and often non-functional proteins. As part of the RNA surveillance, nonsense-mediated decay efficiently eliminates any mRNA that harbors nonsense mutations [13]. For example, loss of tumor suppressor genes have been recognized as a key mechanism in many cancers [14]. Retaining one functional allele of critical genes is essential for survival. Still, null alleles are common: the blood group O is a widely recognized and clinically relevant example [15]. Rare null phenotypes of blood groups have been used to identify null alleles in large populations using routine clinical methods [16,17].

We wondered if the codon usage bias in organisms is associated with a propensity of acquiring nonsense mutations. The consequence of a single nucleotide substitution, like a synonymous, missense or nonsense mutation, is intrinsic in the genetic code. Based on this association, we developed a method to calculate the risk of acquiring nonsense mutations in coding sequences (CDS) relative to an unbiased random codon usage. We applied this method to investigate the codon usage in the whole genome sequences of 40 eukaryotic species.

## Methods

### Risk of acquiring nonsense mutations

**Figure 1:**
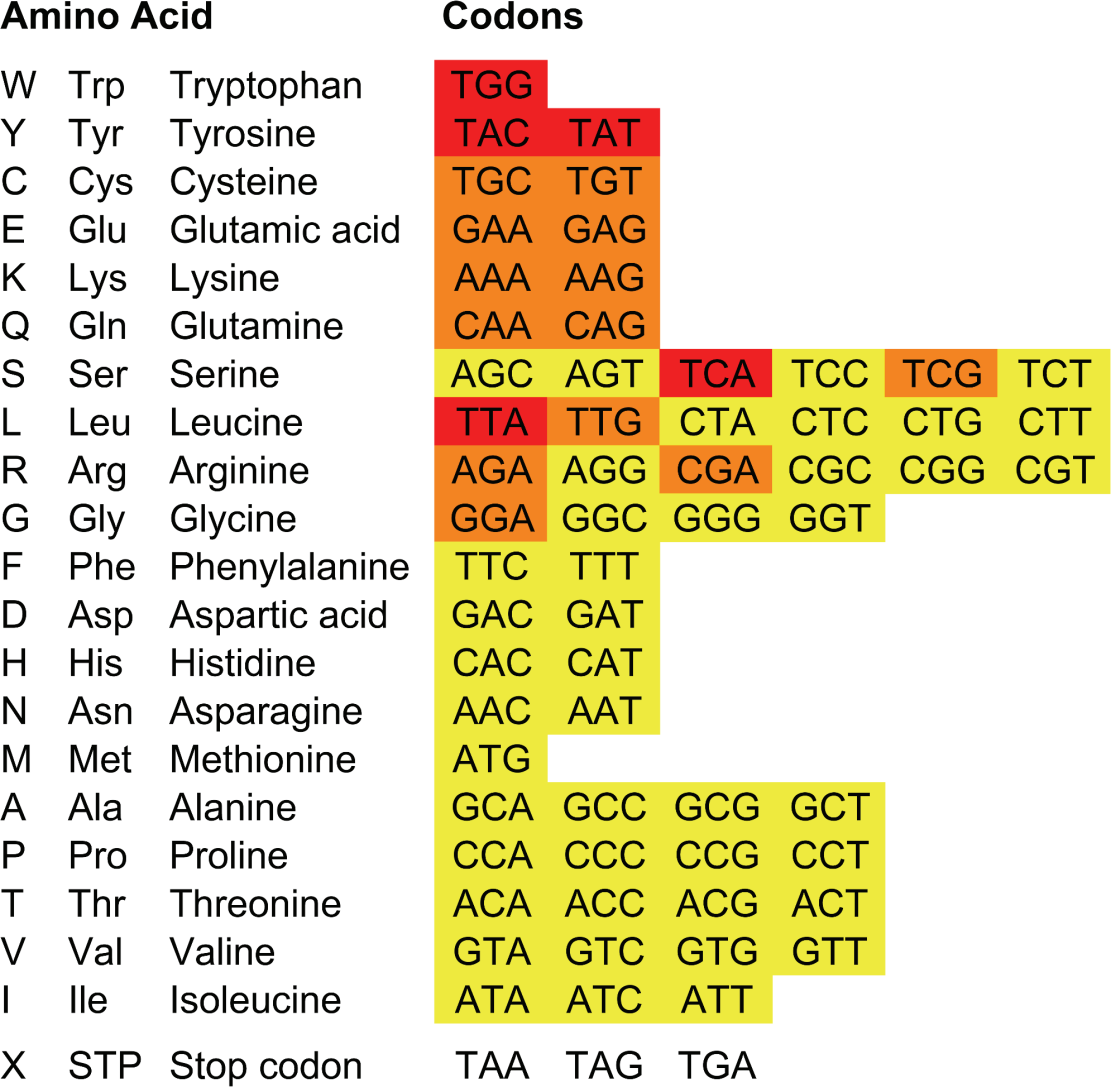
**Genetic code and risk of acquiring nonsense mutations**. The codons of the standard genetic code are listed along with the 20 amino acids and the three stop codons. A risk score *ω *is shown as *ω *= 0 (yellow), *ω *= 1 (orange), and *ω *= 2 (red). The list is sorted according to the mean risk of the codons encoding a specific amino acid.

We used an event based model to estimate the risk of acquiring nonsense mutations by a single nucleotide substitution. A score *ω *of {0, 1, 2} was determined for each of the 61 non-termination codons based on the number of possible single nucleotide substitutions that lead to a stop codon (Figure 1). For this study, the count *c_xxx _*and risk score *ω_xxx _*of each codon *xxx*, with *x *of {A, C, G, T}, was used to determine a risk score *Ω_sequence _*for all coding sequences (CDS) of a species:(1)

To account for the different proteins encoded by the genomes of different species, *Ω_random _*was calculated for comparison assuming an unbiased usage of codons, which was deduced by the number of amino acids *aa_(xxx) _*encoded by codon *xxx *and synonymous codons, and the number of codons encoding this amino acid *n_synonymous,(xxx)_*:(2)

Based on these equations, the parameter "stop risk factor" *F *was calculated for the entire set of CDS in the species' genome:(3)

This *F *defines the risk of acquiring nonsense mutations for each species relative to the risk with an unbiased codon usage. With the intention to compare the risk of acquiring nonsense mutations among various species, we concluded that a random codon usage was the most neutral denominator. These calculations allowed a novel approach to study codon usage bias in whole genomes.

### GC and CpG contents

GC content was calculated as C+G per total nucleotide count, and CpG content as number of CpG dinucleotides per total nucleotide count. The CpG content of genomes was comparable to the results of a recent in silico study [18] for *Pan troglodytes, Mus musculus, Rattus norvegicus, Bos taurus, Canis lupus familiaris*, and *Danio rerio*. Our calculated figures for CpG content match the data obtained by the original in vitro method [19,20].

The expected GC content for the CDS was calculated with the number of codons *n *in the CDS and *GC content_xxx _*denoting the GC content of the codon *xxx*:(4)

The expected CpG content was calculated as described [21]:(5)

### Database and species selection

The NCBI table Eukaryotic Genome Sequencing Projects (March 30, 2010) [22] was used to include all species with a genome status "complete" or "assembly" and an available RefSeq. We restricted analysis to one species per genus (Additional file 1, Figure S1 and Table S1). Sequence data represent NCBI RefSeq database release 40 (March 2010) for 39 species plus GRCh37.p2 (August 2010) for the human genome [23].

### Software

We developed a script driven software package, which parsed the genomic data (FASTA for nucleotide sequences and GenBank flatfile for meta-data including CDS definitions) and calculated the parameters defined in this study, in particular the stop risk factor *F*. In total, 145 GB of data were analyzed.

### Algorithms

**(i) Data selection**. The whole genomes of the species were scanned by the software. Non-standard code sequences, in particular mitochondrial sequences, were excluded from analysis. **(ii) Analysis of the whole genomes**. Nucleotide count, GC content and CpG content were calculated for the genomic sequences of the analyzed species. Non-ACGT nucleotides (3.8%) were excluded. **(iii) Analysis of CDS**. CDS were used as defined in the RefSeq [23]. CDS were excluded that were incomplete at their 5' or 3' end (4.2%) or contained errors (non-triplets 1.3%, no stop codon 0.5%, non-ACGT nucleotides 0.4%). If CDS were associated with an identical geneID, like in splice variants, the longest CDS was used and the alternate sequences (multiples, 13.0%) excluded (Additional file 1, Table S2). *F*, GC content, CpG content and relative codon collection usage were calculated for the CDS.

### Statistical analysis

Results are shown as mean and standard deviation (mean ± SD) or 95% confidence interval (CI) based on the normal distribution, which was tested by D'Agostino-Pearson. We evaluated correlations by Pearson's correlation coefficient *r *and compared the GC content of CDS and genomes among species groups by two-sided Mann-Whitney *U *test. *P *< 0.05 was considered statistically significant. Statistical analysis was done with MedCalc (MedCalc Software, Mariakerke, Belgium).

## Results and Discussion

We analyzed the whole genomes and CDS of 40 eukaryotes (Additional file 1, Tables S1 to S4) to determine the stop risk factor *F *using the propensity of each codon to acquire a nonsense mutation (Figure 1).

### Risk *F *of acquiring nonsense mutations

**Figure 2:**
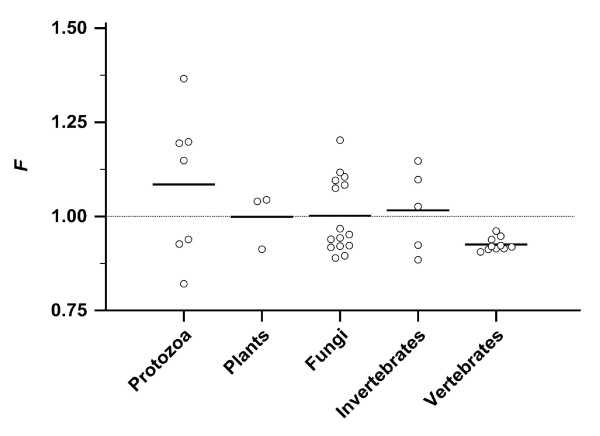
**Stop risk factor *F *in the coding sequences (CDS) of 40 species**. *F *characterizes the relative risk of acquiring nonsense mutations and is shown for 40 species in 5 groups. The black bar represents the mean. The CDS in a species with an unbiased codon usage has an *F *= 1.0 (dotted line).

*F *deviated from the risk of an unbiased codon usage, which is represented by *F *= 1.0 (Figure 2). All 10 vertebrates had an *F *< 1.0 and were clustered (0.93 ± 0.02, range 0.91 - 0.96), while the *F *of all 30 non-vertebrates was higher and ranged widely (1.02 ± 0.13, range 0.82 - 1.37; *P *= 0.019). Fifteen non-vertebrate species had an *F *> 1.0.

### *F *and GC content

**Figure 3:**
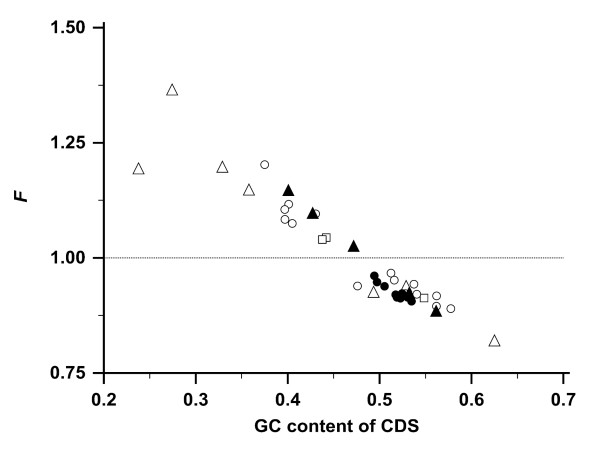
**GC content of CDS relative to *F***. The correlations are shown between the GC content of all CDS in 40 species and the stop risk factor *F*. The species are grouped like in Figure 2: protozoa (△), plants (□), fungi (○), invertebrates (▲), and vertebrates (●). The CDS in a species with an unbiased codon usage has an *F *= 1.0 (dotted line).

*F *correlated strongly and inversely with the GC content of the CDS (Figure 3; Pearson's *r *= -0.95; *P *< 0.0001). The inverse correlation of *F *and GC content is explained by the nucleotide composition of the three stop codons: TAA, TAG, and TGA. The GC content of these three codons is only ^2^/_9_, while the expected mean is ^1^/_2_. Codons with a high GC content have a nucleotide composition that greatly differs from those of stop codons. In comparison, codons with a low GC content are more similar to the stop codons. Hence, codons with a high GC content have on average a lower risk of acquiring a nonsense mutation (Additional file 1, Table S5).

The GC content of codons correlates with the overall GC content of the genomes in many species [9,12,24]. This was confirmed by our data (Additional file 1, Tables S3 and S4). Genes and gene families occur more frequently in genome regions with a high GC content [25,26]. Both observations have been attributed to mechanisms that enrich the GC content, e.g. the increased recombination rates in GC rich regions [27]. High GC content is also associated with increased gene density [28,29], shorter introns [26,28], and longer exons [30].

However, CpG hypermutability, a tenfold increased mutation risk at the position of CpG dinucleotides, causes genomes to drift from a high GC content to a high AT content [31,32]. Active cellular processes are therefore needed to maintain a high GC content [33]. Silencing of specific repair enzymes in *S. typhimurium *strains increases the mutation rate 6-fold to 100-fold with 98% of the mutations converting GC to AT; organisms with AT rich genomes have been explained by the lack of these repair enzymes [34]. Despite knowing several mechanisms to increase and maintain a high GC content in a genome, the utility of a high GC content for an organism is not obvious. The maintenance of a high GC content costs energy and inflicts CpG hypermutability, but is associated with a low risk of acquiring nonsense mutations.

### *F *and CpG content

**Figure 4:**
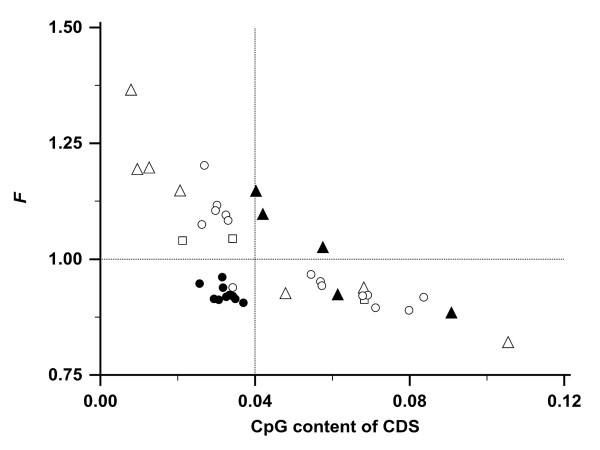
**CpG content of all CDS in 40 species relative to *F***. Symbols are identical to Figure 3: protozoa (△), plants (□), fungi (○), invertebrates (▲), and vertebrates (●). The CDS in a species with an unbiased codon usage has an *F *= 1.0 (horizontal dotted line). All vertebrates have a CpG content < 0.04 (vertical dotted line).

The genomes of all 10 vertebrates had a low risk of acquiring nonsense mutations - as shown by a low *F *- while maintaining a low CpG content along with a low CpG hypermutability (Figure 4). This observation is counterintuitive: low *F *correlated generally with a high GC content (Figure 3) and the associated high CpG content typically inflicts a high risk for mutations. However, all 10 vertebrates expressed a high GC content while keeping the CpG content low in their CDS. The ratio of observed and expected CpG content was lower in the 10 vertebrates (mean 0.48, 95% CI 0.45 - 0.51) than in the 30 non-vertebrates (mean 0.82, 95% CI 0.74 - 0.89; *P *= 0.0001). With the single exception of the fungus *E. cuniculi *(*F *= 0.94 and CpG content = 0.034), harboring the smallest genome in this study, all other 29 non-vertebrate species were exposed either to a high *F *or to a high CpG content in their CDS (Figure 4).

### *F *and codon usage

**Figure 5:**
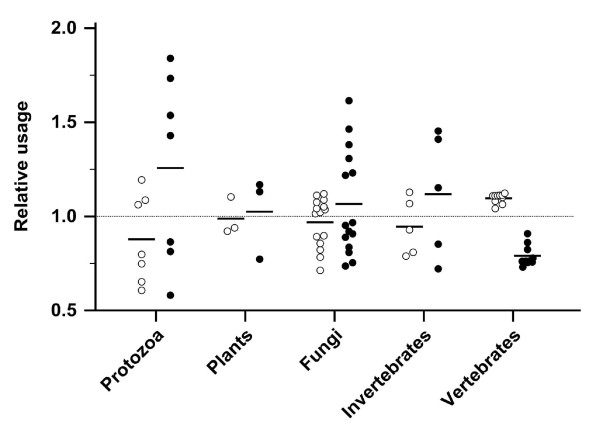
**Relative codon usage for amino acids that can be encoded by codons of various ω (○ for codons with ω = 0; ● for codons with ω = 1 or ω = 2)**. The usage of these codons is shown relative to a random codon usage of 1.0 (dotted line).

In the 10 vertebrates, codon usage was consistently biased towards codons without risk of acquiring nonsense mutations (Figure 5). Codon usage bias can control translation speed and protein folding, increase the efficiency of protein synthesis [1], and be influenced by tRNA concentrations in many species [8]. Nonsense errors that occur during translation delay protein synthesis and cost energy [35]. Use of specific codons is crucial near splice sites because even synonymous mutations at splice sites can lead to splice variants causing phenotypical changes [36] or diseases [37]. The preferred usage of codons with lower risk of acquiring nonsense mutations may indicate an additional driving force for codon usage bias at the genomic level. Indeed, this was found in all vertebrates.

## Conclusions

We show that the codon usage bias in genomes of high GC content is associated with a low risk of acquiring nonsense mutations. Despite their high GC content, the 10 vertebrate genomes had a low CpG content of < 0.04 (Figure 4). The low risk of acquiring nonsense mutations combined with a low exposure to CpG hypermutability [38] is unique in vertebrates. It was not a common feature in the 30 examined non-vertebrates. A low risk of acquiring nonsense mutations may have advantages for organisms with relatively long lifespans and small numbers of offspring.

Calculating *F *is a novel tool for addressing codon usage bias in genes and genomes. Here we applied this approach for comparing the whole genomes among species. *F *can be applied to study GC content shift within the genome of one species [10]. *F *should also provide novel insights in the analysis of individual genes, like oncogenes and evolutionary conserved genes. Based on the fact that a very low *F *indicates a gene with a low risk of acquiring nonsense mutations, *F *may be used as a screening tool among the genes with presently unknown function. First, genes with a very low *F *may more likely belong to the set of crucial genes, whose loss is deleterious for an organism. Second, genes with a very high *F *may have a large number of null alleles in the population, which allows a wider variety of recessive alleles to become phenotypically expressed. Third, the fitness of a species is not just influenced by mutations in its germ line but also in the organism's somatic cells, which could be evaluated using our novel method.

We restricted our current approach to nonsense mutations. It is feasible to broaden our technique and to encompass missense mutations. While nonsense mutations are a more stringent criterion than missense mutations, more codon usage bias could be explained by including unfavorable non-conservative missense mutations in the analysis.

## Conflict of interest disclosure

The authors declare that they have no competing interests.

## Authors' contributions

WAF conceived the study; PS developed the analysis software; WAF and PS analyzed and interpreted the data, and wrote the manuscript. Both authors read and approved the final manuscript.

## Supplementary Material

Additional file 1 **Figure S1. Flowchart for selection of whole genome data sets**. **Table S1**. List of species that were analyzed in this study. **Table S2**. CDS selection for analysis. **Table S3**. CDS analysis data. **Table S4**. Whole genome analysis data. **Table S5**. GC content and risk score ω of the 61 codons. Click here for file
