# Supplementary material for: Codon usage in vertebrates is associated with a low risk of acquiring nonsense mutations
Source: J Transl Med. 2011 Jun 8;9:87. doi: 10.1186/1479-5876-9-87 (PMC3123582; doi:10.1186/1479-5876-9-87)
Supplement: Additional file 1 — Figure S1. Flowchart for selection of whole genome data sets. Table S1. List of species that were analyzed in this study. Table S2. CDS selection for analysis. Table S3. CDS analysis data. Table S4. Whole genome analysis data. Table S5. GC content and risk score ω of the 61 codons. [file 1479-5876-9-87-S1.PDF]

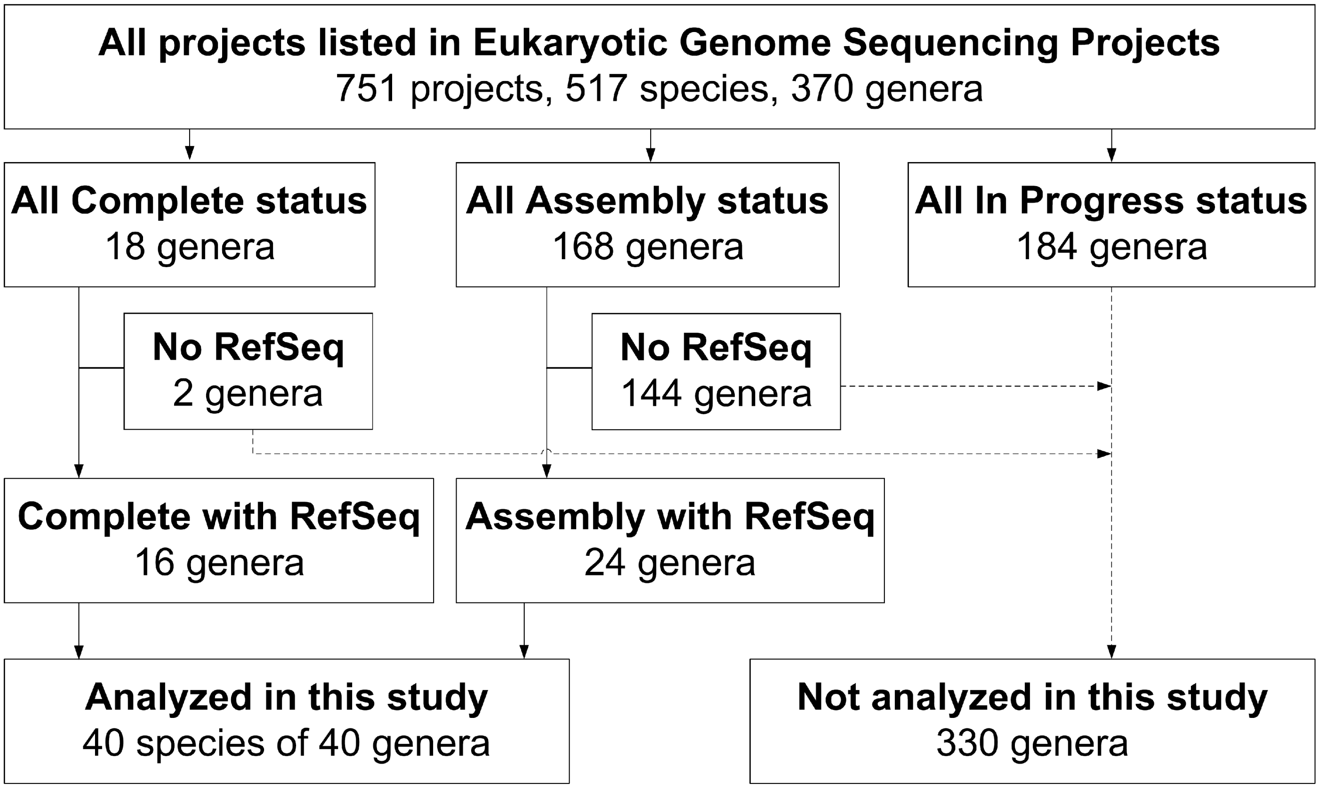

Figure S1. Flowchart for selection of whole genome data sets. We analyzed the species with Complete or Assembly status and accessible RefSeq listing in NCBI Eukaryotic Genome Sequencing Projects as of March 30, 2010. Projects with In Progress status were excluded to avoid any possible bias due to the lack of data for these species.

Table S1. List of species that were analyzed in this study

Group	Species	Accession Type	Nucleotide count
Protozoa	*Cryptosporidium hominis TU502*	NW_	8743570
Protozoa	*Dictyostelium discoideum AX4*	NC_	33943072
Protozoa	*Giardia lamblia ATCC 50803*	NW_	11213615
Protozoa	*Leishmania major strain Friedlin*	NC_	32816778
Protozoa	*Plasmodium falciparum 3D7*	NC_	22856959
Protozoa	*Theileria annulata strain Ankara*	NC_	8352520
Protozoa	*Trypanosoma cruzi strain CL Brener*	NW_	89937456
Plants	*Arabidopsis thaliana*	NC_	119300826
Plants	*Oryza sativa Japonica Group*	NC_	370926643
Plants	*Populus trichocarpa*	NC_	307997801
Fungi	*Ashbya gossypii ATCC 10895*	NC_	8742384
Fungi	*Aspergillus fumigatus Af293*	NC_	29384958
Fungi	*Candida glabrata CBS 138*	NC_	12280357
Fungi	*Cryptococcus neoformans var. neoformans JEC21*	NC_	19051922
Fungi	*Debaryomyces hansenii CBS767*	NC_	12220823
Fungi	*Encephalitozoon cuniculi GB-M1*	NC_	2497519
Fungi	*Gibberella zeae PH-1*	NT_	36258356
Fungi	*Kluyveromyces lactis NRRL Y-1140*	NC_	10689156
Fungi	*Magnaporthe grisea 70-15*	NW_	41624340
Fungi	*Neurospora crassa OR74A*	NW_	39225835
Fungi	*Pichia stipitis CBS 6054*	NC_	15441179
Fungi	*Saccharomyces cerevisiae S288C*	NC_	12070897
Fungi	*Schizosaccharomyces pombe 972h-*	NC_	12571820
Fungi	*Ustilago maydis 521*	NW_	19683350
Fungi	*Yarrowia lipolytica CLIB122*	NC_	20502981
Invertebrates	*Anopheles gambiae str. PEST*	NT_	206073549
Invertebrates	*Apis mellifera DH4*	NW_	236987995
Invertebrates	*Caenorhabditis elegans Bristol N2*	NC_	100267633
Invertebrates	*Drosophila melanogaster*	NT_	96606862
Invertebrates	*Tribolium castaneum Georgia GA2*	NW_	160448733
Vertebrates*	*Danio rerio*	NW_	1440097313
Vertebrates*	*Gallus gallus*	NW_	1078720066
Mammals*	*Bos taurus*	NW_	2906440987
Mammals*	*Canis lupus familiaris*	NW_	2411383226
Mammals*	*Homo sapiens*	GRCh37.p2	3123118147
Mammals*	*Macaca mulatta*	NW_	3011935715
Mammals*	*Monodelphis domestica*	NW_	3571225998
Mammals*	*Mus musculus*	NW_	2569989814
Mammals*	*Pan troglodytes*	NW_	2977702448
Mammals*	*Rattus norvegicus BN/SsNHsdMCW*	NW_	2812707993

* In this study, “vertebrates” denote the 8 mammals plus *D. rerio* and *G. gallus*.

Table S2. CDS selection for analysis

Species	Total CDS	Reason for CDS exclusion					Analyzed CDS
		Multiples	Non-triplets	No stop codon	Incomplete at 5' or 3' end	Non-ACGT nucleotides	
C. hominis	3885	0 (0%)	0 (0%)	0 (0%)	172 (4.4%)	100 (2.6%)	3613 (93%)
D. discoideum	13143	24 (0.2%)	50 (0.4%)	19 (0.1%)	105 (0.8%)	15 (0.1%)	12930 (98.4%)
G. lamblia	6502	0 (0%)	0 (0%)	0 (0%)	1 (0%)	0 (0%)	6501 (100%)
L. major	8142	0 (0%)	0 (0%)	0 (0%)	0 (0%)	2 (0%)	8140 (100%)
P. falciparum	5269	9 (0.2%)	2 (0%)	3 (0.1%)	8 (0.2%)	4 (0.1%)	5243 (99.5%)
T. annulata	3792	0 (0%)	0 (0%)	1 (0%)	4 (0.1%)	1 (0%)	3786 (99.8%)
T. cruzi	19607	0 (0%)	0 (0%)	0 (0%)	3068 (15.6%)	0 (0%)	16539 (84.4%)
A. thaliana	33200	6029 (18.2%)	28 (0.1%)	0 (0%)	4 (0%)	4 (0%)	27135 (81.7%)
O. sativa	26777	0 (0%)	157 (0.6%)	63 (0.2%)	3107 (11.6%)	12 (0%)	23438 (87.5%)
P. trichocarpa	28504	23 (0.1%)	597 (2.1%)	603 (2.1%)	3209 (11.3%)	20 (0.1%)	24052 (84.4%)
A. gossypii	4714	0 (0%)	0 (0%)	0 (0%)	5 (0.1%)	23 (0.5%)	4686 (99.4%)
A. fumigatus	9630	0 (0%)	1 (0%)	0 (0%)	6 (0.1%)	0 (0%)	9623 (99.9%)
C. glabrata	5181	0 (0%)	0 (0%)	0 (0%)	16 (0.3%)	0 (0%)	5165 (99.7%)
C. neoformans	6475	201 (3.1%)	0 (0%)	0 (0%)	0 (0%)	12 (0.2%)	6262 (96.7%)
D. hansenii	6316	0 (0%)	1 (0%)	0 (0%)	133 (2.1%)	21 (0.3%)	6161 (97.5%)
E. cuniculi	1996	0 (0%)	0 (0%)	0 (0%)	1 (0.1%)	0 (0%)	1995 (99.9%)
G. zeae	11578	0 (0%)	0 (0%)	0 (0%)	0 (0%)	0 (0%)	11578 (100%)
K. lactis	5327	0 (0%)	0 (0%)	0 (0%)	108 (2%)	0 (0%)	5219 (98%)
M. grisea	12832	0 (0%)	18 (0.1%)	7 (0.1%)	33 (0.3%)	30 (0.2%)	12744 (99.3%)
N. crassa	9841	20 (0.2%)	2 (0%)	1 (0%)	5 (0.1%)	3 (0%)	9810 (99.7%)
P. stipitis	1276	0 (0%)	15 (1.2%)	9 (0.7%)	143 (11.2%)	0 (0%)	1109 (86.9%)
S. cerevisiae	5862	0 (0%)	0 (0%)	0 (0%)	0 (0%)	0 (0%)	5862 (100%)
S. pombe	4993	0 (0%)	0 (0%)	0 (0%)	4 (0.1%)	0 (0%)	4989 (99.9%)
U. maydis	6522	0 (0%)	0 (0%)	0 (0%)	0 (0%)	0 (0%)	6522 (100%)
Y. lipolytica	6448	0 (0%)	0 (0%)	0 (0%)	0 (0%)	0 (0%)	6448 (100%)
A. gambiae	11531	573 (5%)	709 (6.1%)	426 (3.7%)	2981 (25.9%)	4 (0%)	6838 (59.3%)
A. mellifera	9244	127 (1.4%)	290 (3.1%)	194 (2.1%)	825 (8.9%)	23 (0.2%)	7785 (84.2%)
C. elegans	23894	3723 (15.6%)	0 (0%)	0 (0%)	0 (0%)	0 (0%)	20171 (84.4%)
D. melanogaster	17819	6389 (35.9%)	7 (0%)	1 (0%)	45 (0.3%)	0 (0%)	11377 (63.8%)
T. castaneum	9820	72 (0.7%)	50 (0.5%)	25 (0.3%)	130 (1.3%)	53 (0.5%)	9490 (96.6%)
D. rerio	26913	332 (1.2%)	1125 (4.2%)	476 (1.8%)	1413 (5.3%)	123 (0.5%)	23444 (87.1%)
G. gallus	18540	563 (3%)	804 (4.3%)	227 (1.2%)	2163 (11.7%)	101 (0.5%)	14682 (79.2%)
B. taurus	22676	390 (1.7%)	937 (4.1%)	214 (0.9%)	1110 (4.9%)	278 (1.2%)	19747 (87.1%)
C. lupus familiaris	33638	13878 (41.3%)	66 (0.2%)	31 (0.1%)	147 (0.4%)	74 (0.2%)	19442 (57.8%)
H. sapiens	36785	15867 (43.1%)	616 (1.7%)	83 (0.2%)	469 (1.3%)	0 (0%)	19750 (53.7%)
M. mulatta	37988	13873 (36.5%)	300 (0.8%)	164 (0.4%)	3031 (8%)	376 (1%)	20244 (53.3%)
M. domestica	20173	1084 (5.4%)	92 (0.5%)	48 (0.2%)	275 (1.4%)	49 (0.2%)	18625 (92.3%)
M. musculus	25397	1631 (6.4%)	956 (3.8%)	30 (0.1%)	246 (1%)	101 (0.4%)	22433 (88.3%)
P. troglodytes	51291	26064 (50.8%)	236 (0.5%)	168 (0.3%)	1489 (2.9%)	824 (1.6%)	22510 (43.9%)
R. norvegicus	22895	531 (2.3%)	866 (3.8%)	41 (0.2%)	346 (1.5%)	298 (1.3%)	20813 (90.9%)

Table S3. CDS analysis data

Species	*F*	GC content			CpG content			CpG content per GC content	Analyzed codons
		Observed	Expected*	Ratio	Observed	Expected†	Ratio		
C. hominis	1.198	0.33	0.42	0.77	0.013	0.027	0.47	0.038	1650153
D. discoideum	1.366	0.27	0.41	0.67	0.008	0.019	0.42	0.029	6892196
G. lamblia	0.927	0.49	0.48	1.02	0.048	0.061	0.79	0.097	2808139
L. major	0.821	0.62	0.54	1.17	0.105	0.098	1.08	0.169	5131570
P. falciparum	1.195	0.24	0.36	0.66	0.010	0.014	0.67	0.040	3996936
T. annulata	1.148	0.36	0.42	0.85	0.021	0.032	0.64	0.058	2022698
T. cruzi	0.939	0.53	0.51	1.04	0.068	0.070	0.97	0.129	8317327
A. thaliana	1.044	0.44	0.48	0.93	0.034	0.049	0.70	0.077	10999755
O. sativa	0.913	0.55	0.51	1.07	0.068	0.075	0.91	0.125	9607338
P. trichocarpa	1.040	0.44	0.48	0.91	0.021	0.048	0.44	0.049	8843025
A. gossypii	0.923	0.53	0.49	1.09	0.069	0.070	0.99	0.131	2297039
A. fumigatus	0.921	0.54	0.51	1.07	0.068	0.073	0.93	0.125	4735160
C. glabrata	1.075	0.40	0.45	0.90	0.026	0.041	0.64	0.065	2602688
C. neoformans	0.967	0.51	0.51	1.01	0.054	0.066	0.83	0.106	3355890
D. hansenii	1.203	0.37	0.44	0.84	0.027	0.035	0.77	0.072	2850357
E. cuniculi	0.939	0.48	0.46	1.03	0.034	0.057	0.60	0.072	716641
G. zeae	0.952	0.52	0.50	1.03	0.057	0.067	0.86	0.110	5929052
K. lactis	1.117	0.40	0.45	0.88	0.030	0.040	0.75	0.075	2483326
M. grisea	0.890	0.58	0.52	1.12	0.080	0.083	0.96	0.138	5601351
N. crassa	0.895	0.56	0.51	1.10	0.071	0.079	0.90	0.127	4769316
P. stipitis	1.096	0.43	0.45	0.95	0.032	0.046	0.70	0.075	566814
S. cerevisiae	1.105	0.40	0.45	0.88	0.030	0.039	0.76	0.075	2906865
S. pombe	1.084	0.40	0.46	0.86	0.033	0.039	0.84	0.083	2356404
U. maydis	0.918	0.56	0.52	1.08	0.084	0.079	1.06	0.149	3993690
Y. lipolytica	0.943	0.54	0.49	1.10	0.057	0.072	0.79	0.107	3139837
A. gambiae	0.885	0.56	0.49	1.15	0.091	0.079	1.15	0.162	3253598
A. mellifera	1.147	0.40	0.47	0.86	0.040	0.040	1.00	0.100	4217593
C. elegans	1.098	0.43	0.47	0.92	0.042	0.046	0.92	0.098	8391588
D. melanogaster	0.924	0.53	0.49	1.09	0.061	0.071	0.87	0.115	5997457
T. castaneum	1.026	0.47	0.47	1.01	0.058	0.056	1.03	0.122	5220451
D. rerio	0.961	0.49	0.49	1.02	0.031	0.061	0.52	0.064	12614136
G. gallus	0.939	0.51	0.49	1.02	0.032	0.064	0.50	0.063	7444184
B. taurus	0.920	0.52	0.49	1.06	0.034	0.067	0.51	0.066	10003189
C. lupus familiaris	0.906	0.53	0.50	1.07	0.037	0.071	0.52	0.069	10795375
H. sapiens	0.919	0.52	0.50	1.05	0.033	0.068	0.48	0.062	11151493
M. mulatta	0.914	0.53	0.50	1.05	0.035	0.070	0.50	0.066	8998157
M. domestica	0.948	0.50	0.49	1.01	0.026	0.062	0.42	0.052	10020218
M. musculus	0.915	0.52	0.49	1.05	0.029	0.067	0.44	0.057	10081664
P. troglodytes	0.923	0.52	0.50	1.04	0.033	0.069	0.49	0.064	9628671
R. norvegicus	0.912	0.52	0.50	1.05	0.031	0.068	0.45	0.059	9864903

* The expected GC content is calculated based on a random codon usage.

† The expected CpG content is calculated based on the actual GC content.

Table S4. Whole genome analysis data

Species	Observed GC content	CpG content			CpG content per GC content
		Observed	Expected*	Ratio	
C. hominis	0.31	0.012	0.024	0.52	0.040
D. discoideum	0.22	0.007	0.013	0.52	0.029
G. lamblia	0.49	0.047	0.061	0.77	0.095
L. major	0.60	0.091	0.089	1.02	0.152
P. falciparum	0.19	0.007	0.009	0.76	0.037
T. annulata	0.33	0.017	0.026	0.65	0.053
T. cruzi	0.52	0.065	0.067	0.97	0.125
A. thaliana	0.36	0.023	0.032	0.72	0.065
O. sativa	0.44	0.041	0.047	0.87	0.095
P. trichocarpa	0.33	0.015	0.028	0.52	0.044
A. gossypii	0.52	0.067	0.067	1.00	0.130
A. fumigatus	0.50	0.054	0.062	0.86	0.107
C. glabrata	0.39	0.025	0.037	0.66	0.064
C. neoformans	0.49	0.050	0.059	0.85	0.103
D. hansenii	0.36	0.026	0.033	0.80	0.073
E. cuniculi	0.47	0.034	0.056	0.60	0.071
G. zeae	0.48	0.049	0.058	0.83	0.101
K. lactis	0.39	0.029	0.038	0.77	0.075
M. grisea	0.52	0.062	0.067	0.92	0.119
N. crassa	0.49	0.055	0.061	0.91	0.112
P. stipitis	0.41	0.031	0.042	0.73	0.075
S. cerevisiae	0.38	0.029	0.037	0.80	0.077
S. pombe	0.36	0.029	0.033	0.89	0.080
U. maydis	0.54	0.077	0.073	1.05	0.142
Y. lipolytica	0.49	0.048	0.060	0.81	0.099
A. gambiae	0.44	0.053	0.049	1.07	0.119
A. mellifera	0.33	0.044	0.027	1.66	0.136
C. elegans	0.35	0.031	0.031	0.99	0.088
D. melanogaster	0.42	0.042	0.045	0.92	0.098
T. castaneum	0.34	0.033	0.029	1.16	0.098
D. rerio	0.37	0.018	0.033	0.53	0.049
G. gallus	0.42	0.011	0.043	0.25	0.026
B. taurus	0.42	0.010	0.044	0.23	0.024
C. lupus familiaris	0.41	0.011	0.043	0.26	0.027
H. sapiens	0.41	0.009	0.041	0.24	0.024
M. mulatta	0.41	0.010	0.042	0.25	0.026
M. domestica	0.38	0.005	0.036	0.13	0.013
M. musculus	0.42	0.008	0.043	0.19	0.020
P. troglodytes	0.41	0.010	0.041	0.24	0.024
R. norvegicus	0.42	0.010	0.044	0.22	0.023

* The expected CpG content is calculated based on the actual GC content.

Table S5. GC content and risk score ω of the 61 codons

	Number of codons							
	encoding amino acids represented by variable ω				encoding amino acids represented by identical ω			
GC content	0	1	2	Total codons	0	1	2	Total codons
0	0	0	1	1	4	1	1	6
$^{1}/_{3}$	4	2	1	7	10	4	1	15
$^{2}/_{3}$	7	3	0	10	10	3	1	14
1	4	0	0	4	4	0	0	4
Total codons	15	5	2	22	28	8	3	39
